# Epigenetic regulation of CD8^+^ T cell exhaustion: recent advances and update

**DOI:** 10.3389/fimmu.2025.1700039

**Published:** 2025-10-21

**Authors:** Chunhong Li, Yixiao Yuan, Xiulin Jiang, Qiang Wang

**Affiliations:** ^1^ Department of Oncology, Suining Central Hospital, Suining, Sichuan, China; ^2^ Department of Systems Biology, City of Hope Comprehensive Cancer Center Biomedical Research Center, Monrovia, CA, United States; ^3^ Department of Gastrointestinal Surgical Unit, Suining Central Hospital, Suining, Sichuan, China

**Keywords:** CD8+ T cell exhaustion, epigenetic regulation, DNA methylation, histone modification, transcription factors, immunotherapy

## Abstract

CD8^+^ T cells play a pivotal role in antiviral and antitumor immunity, yet under chronic antigen stimulation, they progressively enter a functionally impaired “exhausted” state, characterized by loss of effector functions, sustained high expression of inhibitory receptors, and a distinct transcriptional and epigenetic landscape. Recent studies have highlighted that epigenetic regulation is central to the initiation and maintenance of CD8^+^ T cell exhaustion. Exhausted T cells exhibit chromatin landscapes markedly different from those of effector and memory T cells, displaying an “epigenetic locking” that renders their phenotype largely irreversible. Emerging evidence highlights the central role of epigenetic and transcriptional regulation in driving and maintaining CD8^+^ T cell exhaustion. DNA methylation and histone modifications establish stable repressive chromatin landscapes that suppress effector gene programs. Non-coding RNAs, including microRNAs and long non-coding RNAs, fine-tune exhaustion-associated pathways post-transcriptionally, while RNA epigenetic modifications, such as m6A methylation, regulate transcript stability and translation in exhausted T cells. Transcription factors orchestrate these epigenetic and post-transcriptional networks, reinforcing exhaustion-specific gene expression profiles. Together, these interconnected mechanisms not only define the exhausted phenotype but also contribute to tumor immune evasion and therapeutic resistance. Understanding these processes provides a framework for novel strategies aimed at reversing CD8^+^ T cell exhaustion and improving the efficacy of cancer immunotherapy. Collectively, elucidating the epigenetic mechanisms underlying CD8^+^ T cell exhaustion not only deepens our understanding of its molecular basis but also provides new avenues for precision immunotherapy and individualized interventions.

## 1 Introduction

CD8^+^ T cells are key effector lymphocytes responsible for eliminating intracellular pathogens and malignant cells (1). During acute infections or immunogenic tumors, antigen recognition via the T cell receptor (TCR) drives rapid proliferation and differentiation into effector populations with potent cytotoxicity and cytokine secretion capacity, followed by the formation of a long-lived memory pool upon resolution (2). However, under conditions of chronic antigen exposure—such as persistent viral infections (HBV, HCV, HIV) or the tumor microenvironment (TME)—CD8^+^ T cells progressively enter a hierarchically impaired state termed T cell exhaustion (3). This state is characterized initially by a decline in cytokine production (e.g., IL-2, TNF-α), followed by reduced cytotoxicity and proliferative potential, alongside sustained high expression of inhibitory receptors including PD-1, TIM-3, LAG-3, and TIGIT, accompanied by distinct transcriptional, metabolic, and epigenetic landscapes (3). Importantly, exhaustion is mechanistically and phenotypically distinct from anergy and senescence: it is driven by persistent antigen, exhibits graded functional deficits, and possesses unique molecular signatures, whereas anergy and senescence differ in triggers, molecular trajectories, and reversibility (4).

Recent single-cell and lineage-tracing studies have further delineated heterogeneity within the exhausted T cell pool, identifying progenitor-like exhausted cells (TCF1^+^/PD-1^+^) and terminally exhausted cells (PD-1^hi^/TOX^hi^) with distinct roles in sustained responses, therapeutic responsiveness, and fate determination (5). In chronic viral infections, exhaustion limits viral clearance and correlates with disease progression; in tumors, persistent antigen load, combined with multiple suppressive signals within the TME—including TGF-β, IL-10, adenosine, hypoxia, and lactate accumulation—drives both the induction and maintenance of exhaustion (6). Clinically, immune checkpoint inhibitors (ICIs) can partially alleviate functional impairment, particularly when a significant fraction of progenitor-like exhausted T cells is preserved, whereas dominance of terminal exhaustion often predicts resistance or suboptimal responses (7). These observations suggest that merely releasing inhibitory receptor signaling may be insufficient for durable functional restoration, and deeper molecular mechanisms must be addressed.

Epigenetic regulation serves as a central hub in establishing and stabilizing the exhausted phenotype (8). Compared with effector and memory T cells, exhausted T cells display broadly remodeled and stable chromatin accessibility and enhancer networks: loci encoding inhibitory receptors and exhaustion-associated transcription factors (e.g., TOX, NR4A family) remain persistently open, whereas effector molecules (IFN-γ, GZMB) and metabolic adaptation loci are relatively closed (9). At the DNA methylation level, inhibitory pathway genes such as *PDCD1* are hypomethylated and transcriptionally active, while effector program loci undergo remethylation-mediated repression (6). Histone modifications also exhibit distinct patterns, with repressive marks (e.g., H3K27me3) enriched at effector gene loci and activating marks (H3K4me1, H3K27ac) concentrated at exhaustion-associated regulatory elements (6). Importantly, transcription factor–epigenetic coupling forms a positive feedback loop: NFAT, BATF, TOX, and NR4A not only drive the exhaustion transcriptional network but also recruit chromatin-modifying complexes to reinforce this landscape, constituting an “epigenetic lock (6).” Furthermore, RNA epigenetic modifications, such as m^6^A, fine-tune mRNA stability and translation efficiency, influencing T cell differentiation trajectories and functional maintenance, adding an additional regulatory layer to exhaustion (10). Epigenetic regulation offers unique therapeutic opportunities in modulating CD8^+^ T cell exhaustion because, unlike genetic modification, it does not involve permanent changes to the DNA sequence, allowing reversible and dynamic control of gene expression (11). Compared with metabolic interventions, which can have broad systemic effects, epigenetic therapies can more selectively reprogram T cell fate by targeting specific chromatin states, histone marks, or DNA/RNA modifications (12). This precision enables the restoration of effector functions in exhausted T cells while minimizing off-target effects, making epigenetic strategies particularly attractive for immunotherapeutic applications.

Based on these insights, investigating the epigenetic regulation of CD8^+^ T cell exhaustion holds significant translational implications. First, it enables identification of novel therapeutic targets (e.g., DNA methyltransferases, HDACs, EZH2, BET family proteins) and optimization of combination strategies (epigenetic drugs plus ICIs) to achieve “partial unlocking” of exhaustion and functional recovery within safe limits (13). Second, multi-omics biomarkers—including ATAC-seq, methylome, and histone modification profiling—can predict immune therapy responsiveness and resistance risk, allowing dynamic stratification across patient populations and time. Third, these principles can guide rational engineering of T cells (e.g., via gene and epigenetic editing) to enhance anti-exhaustion capacity, thereby improving the durability and efficacy of adoptive cell therapies (14). Collectively, a systematic elucidation of the “epigenetic–exhaustion axis” provides a robust conceptual and methodological foundation for understanding CD8^+^ T cell exhaustion and enhancing antiviral and antitumor immunity.

While T cell exhaustion has been extensively reviewed, including its epigenetic regulation (Belk, 2022), the rapidly expanding landscape of multi-omic technologies and mechanistic insights warrants an updated synthesis. In this mini-review, we focus specifically on the integration of epigenetic, transcriptional, and RNA-level regulatory mechanisms that collectively govern CD8^+^ T cell exhaustion, highlighting novel layers of control that extend beyond classical DNA methylation and histone modifications. We emphasize emerging findings on RNA epigenetic modifications non-coding RNAs, and transcription factor-mediated chromatin remodeling, and how these intersect to reinforce exhaustion phenotypes in chronic infection and cancer. Additionally, we discuss recent technological advances in single-cell multi-omics, epigenome editing, and functional perturbation studies, providing a framework for precision immunotherapeutic strategies. By doing so, this review complements and extends prior work by explicitly linking these mechanistic insights to potential avenues for reversing exhaustion and improving cancer immunotherapy outcomes.

## 2 Epigenetic features of exhausted T cells

**Figure 1:**
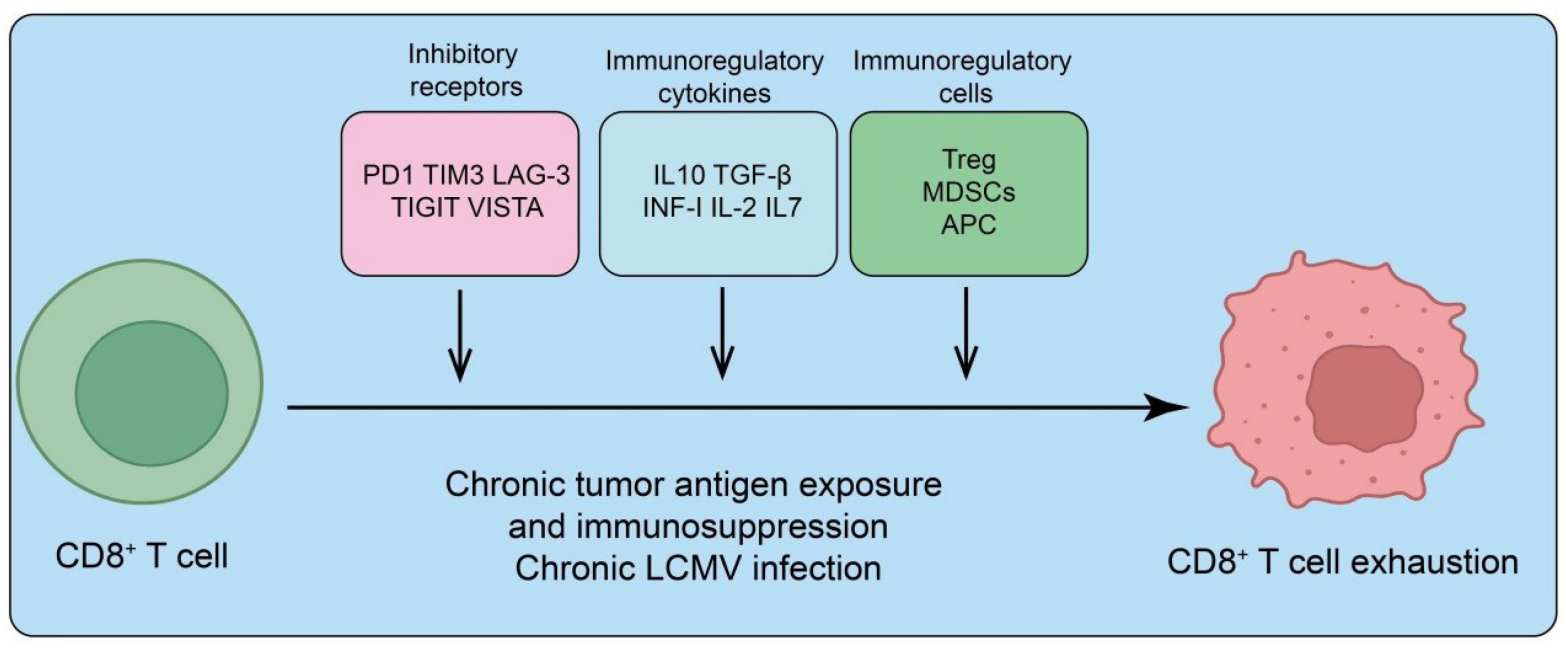
Mechanisms driving CD8^+^ T cell exhaustion. Chronic tumor antigen exposure or persistent infection (e.g., chronic LCMV) promotes CD8^+^ T cell dysfunction through multiple immunosuppressive pathways. Upregulation of inhibitory receptors (PD-1, TIM-3, LAG-3, TIGIT, VISTA) dampens T cell receptor signaling. Immunoregulatory cytokines (IL-10, TGF-β, type I interferons, IL-2, IL-7) further suppress effector function and sustain an exhausted state. In addition, immunoregulatory cell populations, including regulatory T cells (Treg), myeloid-derived suppressor cells (MDSCs), and antigen-presenting cells (APCs), reinforce immunosuppression and drive progressive CD8^+^ T cell exhaustion.

The hallmark of CD8^+^ T cell exhaustion extends beyond functional and phenotypic alterations, with its core drivers rooted in distinctive epigenetic remodeling (15). Compared with effector or memory T cells, exhausted T cells exhibit extensively restructured chromatin landscapes and stably maintained transcriptional programs. The hallmark of CD8^+^ T cell exhaustion is the sustained high expression of inhibitory receptors (IRs) such as programmed cell death-1 (PD-1), T cell immunoglobulin and mucin domain protein-3 (TIM-3), lymphocyte activation gene-3 (LAG-3), T cell immunoreceptor with immunoglobulin and ITIM domains (TIGIT), V-domain immunoglobulin suppressor of T cell activation (VISTA), and cytotoxic T lymphocyte-associated protein-4, accompanied by reduced secretion of cytokines including interleukin-2 (IL-2), tumor necrosis factor (TNF), and interferon-γ (IFN-γ) (16–18). Exhausted CD8^+^ T cells also exhibit altered expression of genes related to chemotaxis, migration, and adhesion, as well as defects in metabolism and other cellular functions (19, 20) (**Figure 1**). The key molecular and cellular mechanisms that either drive or counteract T-cell exhaustion are summarized in **Table 1**, providing an overview of pathways that influence T-cell fate in chronic infection and cancer. Although IRs can be transiently upregulated during CD8^+^ T cell activation, their sustained high expression is a defining feature of exhaustion. Within exhausted T cells, PD-1 is often co-expressed with TIM-3, LAG-3, CTLA-4, and other IRs; however, the expression of PD-1 or other individual IRs alone does not necessarily indicate CD8^+^ T cell exhaustion (21). In contrast, chromatin regions encompassing effector genes (e.g., IFN-γ, GZMB, IL-2) and metabolic regulators are predominantly closed or repressed, resulting in marked impairment of cytotoxicity, proliferative capacity, and cytokine secretion (22). This coordinated imbalance between function and epigenetic state constitutes the molecular basis for the stable manifestation of the exhausted phenotype. The epigenetic features of exhausted T cells are highly stable and largely irreversible. Even upon removal of extrinsic inhibitory signals—such as PD-1/PD-L1 blockade or tumor antigen clearance—the terminally exhausted state remains difficult to fully reverse. This phenomenon, termed “epigenetic lock-in(Refers to the stable maintenance of a specific gene expression state through heritable chromatin modifications (e.g., DNA methylation, histone marks) that resist reversal, even after removal of the original stimulus. In T cell exhaustion, it denotes the persistent silencing of effector genes due to fixed epigenetic configurations),” reflects the long-term fixation of chromatin accessibility, transcription factor networks, and histone modifications (22). Specifically, progenitor-like exhausted T cells (TCF1^+^/PD-1^+^) retain partial open chromatin regions and possess limited self-renewal and differentiation potential, whereas terminally exhausted T cells (PD-1^hi^/TOX^hi^) exhibit closed chromatin and restricted transcription of effector genes, rendering their functionality nearly irreversible. Single-cell analyses and ATAC-seq further confirm pronounced heterogeneity within the exhausted T cell pool, which not only influences responsiveness to immunotherapies but also indicates that exhaustion represents a dynamic, hierarchically organized epigenetic process rather than a uniform state (23).

**Table 1:** Key molecular and cellular factors promoting or preventing T-cell exhaustion.

Promoting T-cell exhaustion	Preventing / limiting T-cell exhaustion
Chronic antigen stimulation – Persistent viral or tumor antigens sustain TCR signaling, activating NFAT and inducing exhaustion genes (PD-1, TOX).	Co-stimulatory receptors (CD28, ICOS, 4-1BB, OX40) – Provide secondary signals to maintain Akt/mTOR activity and T-cell proliferation.
Inhibitory receptors – PD-1/PD-L1, CTLA-4, LAG-3, TIM-3, TIGIT, BTLA suppress TCR/CD28 signaling and cytokine production.	Supportive transcription factors – TCF-1, Bcl-6 preserve progenitor-like Tex^prog^ cells with self-renewal and responsiveness to checkpoint blockade.
Exhaustion-driving transcription factors – TOX, NR4A family, BATF, Eomes sustain inhibitory receptor expression and exhaustion program.	Homeostatic cytokines – IL-7, IL-15, IL-21 promote memory differentiation and mitochondrial fitness.
Immunosuppressive cytokines – TGF-β, IL-10, VEGF inhibit effector differentiation and foster regulatory phenotypes.	Metabolic enhancers – PGC-1α activation, AMPK signaling increase mitochondrial biogenesis and energy supply.
Metabolic dysregulation – Glucose deprivation, lactic acid accumulation, lipid peroxidation, mTOR inhibition limit glycolysis and ATP generation.	Immune checkpoint blockade – Anti-PD-1, anti-CTLA-4, anti-LAG-3 antibodies relieve inhibitory signaling and restore effector cytokine production.
Suppressive cells in TME – Regulatory T cells (Tregs), myeloid-derived suppressor cells (MDSCs), tumor-associated macrophages (TAMs) secrete IL-10/TGF-β and deplete nutrients (e.g., arginine).	Tumor microenvironment modulation – VEGF inhibition or lactate depletion improves nutrient availability and oxygenation.

T cell exhaustion limits effector T cell function in chronic infections and tumors. Traditionally, the development of exhausted T cells and their precursors has been thought to require sustained antigen exposure and inflammatory stimuli. However, recent studies indicate that similar T cell populations also emerge during the early phase of acute infections (24). At this stage, early-developing TCF1^+^ precursor cells display unexpected heterogeneity, encompassing both precursors of conventional memory T cells and cells with phenotypic, transcriptional, and epigenetic features reminiscent of exhausted T cell precursors observed in chronic infections (24). High-affinity ligand engagement promotes the formation of these precursors, whereas PD-1 signaling restricts their development. Although exhausted precursors are relatively abundant during the early phase of infection, their numbers decline once the immune system resolves the infection, yet they are not entirely eliminated (24). Moreover, the epigenetic remodeling of exhausted T cells involves the coordinated interplay of multiple mechanisms. DNA methylation, histone modifications (e.g., H3K27me3, H3K4me1, H3K27ac), reprogramming of enhancer and silencer elements, and interactions between transcription factors and epigenetic complexes collectively form a stable positive feedback loop. This loop maintains sustained expression of inhibitory receptors and exhaustion-associated transcription factors while restricting activation of effector programs, thereby consolidating the exhausted state (23). In summary, the epigenetic landscape of exhausted T cells integrates chromatin remodeling, functional limitation, epigenetic lock-in, and population heterogeneity. These features not only explain the difficulty in fully reversing exhaustion but also provide a theoretical basis and potential targets for epigenetic interventions—such as DNA methylation inhibitors and histone-modifying enzyme inhibitors—combined with immune checkpoint blockade, offering new strategies to enhance immunity in chronic infections and cancer (25). To provide an integrated overview, **Table 1** summarizes the major epigenetic regulators involved in CD8^+^ T cell exhaustion, their principal molecular targets, and the corresponding functional consequences that shape exhaustion dynamics (**Table 2**).

**Table 2:** Major epigenetic regulators involved in CD8^+^ T cell exhaustion.

Category	Epigenetic regulator	Primary targets / mechanisms	Functional consequences in CD8^+^ T cell exhaustion
DNA methylation	DNMT3A	*De novo* methylation of effector gene promoters (*IFNG*, *GZMB*); stable silencing of memory-associated loci	Reinforces terminal exhaustion and limits reinvigoration after PD-1 blockade
	TET2	Catalyzes 5mC to 5hmC conversion, promoting demethylation of effector and stemness genes	Loss of TET2 enhances Tex stability but reduces effector recall capacity
Histone modifications	EZH2 (PRC2 complex)	Adds H3K27me3 to repress effector genes (*IFNG*, *TBX21*)	Supports differentiation toward terminal Tex phenotype
	HDAC1/2/3	Deacetylation of histones and transcription factors	Reduces chromatin accessibility and effector gene transcription; HDAC inhibition partially reverses exhaustion
	p300/CBP	Histone acetyltransferases that activate enhancers of *IL2*, *GZMB*	Promote effector cytokine production; their loss facilitates exhaustion
Chromatin remodeling complexes	BRG1/SMARCA4 (BAF complex)	ATP-dependent chromatin remodeling at effector and memory loci	Maintains transcriptional accessibility of effector programs
	PBAF complex (PBRM1, ARID2)	Recruits repressive marks at exhaustion-related enhancers	Restricts effector differentiation and reinforces exhaustion stability
Transcriptional–epigenetic regulators	TOX	Recruits chromatin modifiers (HDACs, DNMTs) to exhaustion loci (*PDCD1*, *LAG3*, *TIM3*)	Establishes exhaustion-specific chromatin accessibility; critical for Tex lineage stability
	TCF1 (encoded by TCF7)	Maintains open chromatin at progenitor loci; represses terminal exhaustion genes	Defines progenitor-like Tex subset responsive to PD-1 blockade
RNA epigenetic modification	METTL3	Catalyzes m^6^A methylation of *Tcf7*, *Myc*, and metabolic regulators	Regulates RNA stability and translation; deficiency accelerates exhaustion
	ALKBH5	m^6^A demethylase that removes methyl marks from effector-related transcripts	Sustains effector functions and limits terminal exhaustion
Non-coding RNAs	miR-155 / miR-31 / miR-21	Target transcription factors (*Eomes*, *BATF*) and cytokine signaling pathways	Modulate the balance between effector and exhausted states
Emerging regulators	Histone lactylation	Links glycolytic metabolism to transcriptional activation	May reactivate suppressed effector genes in Tex cells

## 3 DNA methylation and CD8^+^ T cell exhaustion

DNA methylation is a key epigenetic regulatory mechanism that modulates gene transcription by adding methyl groups to promoters or gene bodies, typically at CpG islands. In CD8^+^ T cell exhaustion (Tex), DNA methylation plays a central role in both the establishment and maintenance of the exhausted state (26). Chronically stimulated CD8^+^ T cells acquire a distinctive DNA methylation landscape that differs markedly from effector (Teff) or memory (Tmem) T cells, characterized by methylation of exhaustion-associated loci and silencing of critical effector genes (26). For instance, inhibitory receptor genes such as PD-1 (*PDCD1*), TIM-3, and LAG-3 exhibit specific methylation patterns that sustain their high expression in Tex cells, whereas effector cytokine genes like IL-2 and IFN-γ are often hypermethylated and transcriptionally repressed, leading to functional impairment (26). Studies indicate that DNA methylation not only stabilizes the exhausted phenotype but also confers an “epigenetic memory,” making the exhausted state difficult to reverse even after clearance of chronic infection or tumor antigens (27). This concept aligns with the previously described “epigenetic scar,” highlighting that antigen removal or immunotherapeutic intervention alone may be insufficient to restore T cell function, and targeted modulation of methylation is necessary (9, 28). DNA methylation interacts with other epigenetic mechanisms, including histone modifications and chromatin remodeling, collectively shaping the unique chromatin landscape of Tex cells, thereby rendering exhaustion both stable and largely irreversible (27). In the context of immunotherapy, targeting DNA methylation shows potential translational value. DNA methyltransferase inhibitors can partially remodel the epigenetic state of exhausted T cells, restore effector functions, and enhance immune responses against tumors or chronic infections (29). Moreover, combining DNA methyltransferase inhibitors with ICIs, such as anti-PD-1/PD-L1 antibodies, holds promise for improving therapeutic efficacy, offering new strategies and mechanistic rationale for reversing CD8^+^ T cell exhaustion (30).

**Figure 2:**
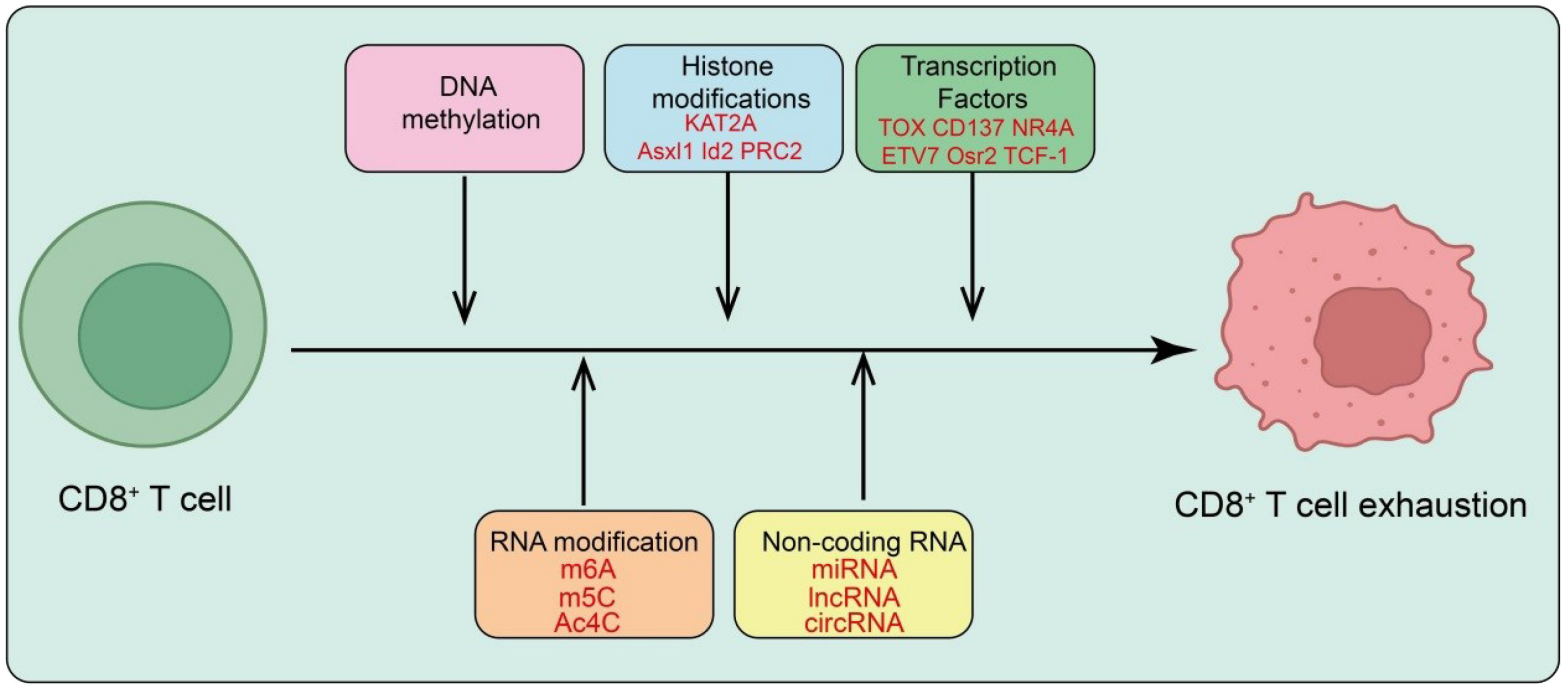
Epigenetic and transcriptional regulation of CD8^+^ T cell exhaustion. Persistent antigen stimulation drives CD8^+^ T cells toward exhaustion through multilayered epigenetic and transcriptional programs. DNA methylation and histone modifications (e.g., KAT2A, Asx1, Id2, PRC2) reshape chromatin to establish an exhaustion-associated landscape. Key transcription factors, including TOX, CD137, NR4A, ETV7, Osr2, and TCF-1, orchestrate gene-expression programs that sustain the dysfunctional state. RNA modifications (m^6^A, m^5^C, Ac^4^C) and non-coding RNAs (miRNA, lncRNA, circRNA) further modulate the epigenetic and transcriptional machinery, reinforcing and stabilizing the exhausted phenotype of CD8^+^ T cells.

Emerging evidence highlights the role of Runx3 methylation in regulating CD8^+^ T cell function. Promoter demethylation of Runx3 promotes infiltration of tumor-infiltrating CD8^+^ T cells and alleviates T cell exhaustion (31). Tissue-specific Runx3 knockout mouse models demonstrate reduced CD8^+^ T cell infiltration, impaired differentiation into effector and memory T cells, and markedly decreased levels of chemokines CCR3 and CCR5 (31). Notably, treatment with decitabine (DAC) combined with anti-PD-1 therapy is ineffective in the absence of Runx3, underscoring its central role in mediating immune responses. In pancreatic ductal adenocarcinoma (PDAC), poor immunotherapeutic efficacy is primarily attributed to an immunosuppressive tumor microenvironment and T cell exhaustion, while the contribution of interferon-stimulated genes remains unclear (32, 33). GBP4, a prominent member of the GBP family in the PDAC microenvironment, shows a negative correlation with patient survival (26). DNA hypomethylation is observed in regulatory regions of GBP4, and its overexpression significantly enhances CD8^+^ T cell infiltration but also upregulates immune checkpoint genes and promotes T cell exhaustion. Ex vivo T cell cytotoxicity assays using primary organoids demonstrate that PDAC samples with high GBP4 expression exhibit markedly increased sensitivity to anti-PD-1 therapy (26). Collectively, DNA methylation represents a pivotal epigenetic mechanism underlying the formation and maintenance of CD8^+^ T cell exhaustion. Its specific regulatory patterns not only explain the stability of the exhausted phenotype but also reveal novel avenues for therapeutic intervention, providing important insights for cancer immunotherapy and the management of chronic infections (**Figure 2**).

## 4 Histone modifications and CD8^+^ T cell exhaustion

Histone modifications and chromatin states are key epigenetic mechanisms governing the formation and maintenance of CD8^+^ Tex (34). Histones undergo chemical modifications—including acetylation, methylation, phosphorylation, and ubiquitination—that regulate chromatin accessibility and gene transcription (34). In Tex cells, histone modifications synergize with chromatin remodeling to establish a unique epigenetic landscape. Repressive histone methylation marks (e.g., H3K27me3) are enriched at promoters of critical effector and memory genes, silencing IL-2, IFN-γ, and other effector loci (35). Conversely, activating marks such as H3K4me3 and H3K27ac are enriched at cis-regulatory elements of exhaustion-associated genes, including PD-1, TIM-3, and LAG-3, promoting sustained high expression of inhibitory receptors (36). These histone modification patterns not only shape Tex-specific transcriptional programs but also confer stability and heritability to the exhausted state. Histone modifications and chromatin states are closely coordinated with transcription factor networks. Transcription factors such as TOX, NR4A, ETV7, and Fli1 maintain exhaustion programs in concert with specific histone modifications and accessible chromatin regions, while signals mediated by CD137 and MGP can influence Tex proliferation and differentiation through chromatin and transcription factor modulation (37). Understanding the regulation of exhaustion by histone modifications and chromatin states has important implications in immunotherapy. Pharmacological targeting of histone-modifying enzymes, such as histone deacetylase inhibitors or histone methyltransferase inhibitors, can enhance chromatin accessibility, restore effector gene expression, and potentiate antitumor activity. Combined with ICIs, these strategies represent promising avenues to reverse CD8^+^ T cell exhaustion and improve antitumor immunity (38).

During chronic infections and cancer, Tex cells undergo metabolic and epigenetic reprogramming, impairing protective functions. Studies show that Tex cells downregulate acetyl-CoA synthetase 2 (ACSS2) while maintaining ATP-citrate lyase (ACLY) activity, shifting from acetate-dependent to citrate-dependent metabolism (34). This metabolic switch enhances citrate-dependent histone acetylation at Tex-specific genes through KAT2A–ACLY interactions while reducing acetate-dependent acetylation (mediated by the p300–ACSS2 complex) at effector and memory gene loci. Functional experiments demonstrate that nuclear ACSS2 overexpression or ACLY inhibition can block Tex differentiation and augment tumor-specific CD8^+^ T cell responses (34). These findings reveal a nutrient-metabolism-guided histone acetylation code, whereby metabolites regulate histone acetylation through specific enzyme complexes to determine CD8^+^ T cell fate, providing a rationale for metabolism–epigenetics–guided T cell therapies. Deletion of Dnmt3a, Tet2, or Asxl1 in CD8^+^ T cells under chronic antigen exposure preserves progenitor-like Tex populations for over a year without malignant transformation (39). Asxl1 regulates histone H2AK119 ubiquitination and polycomb group–repressive deubiquitinase (PR-DUB) pathways to control T cell self-renewal and reduce differentiation (40). Functional studies show that Asxl1-deficient T cells synergize with anti-PD-L1 therapy, enhancing tumor control and conferring survival advantages to mutated T cells (39). In head and neck squamous cell carcinoma, increased histone lactylation correlates with poor immunotherapy response (41). H3K9la is identified as a head and neck squamous cell carcinoma -specific modification (42); CUT&Tag analysis reveals IL-11 as a downstream target. IL-11 activates immune checkpoint gene transcription via the JAK2/STAT3 pathway in CD8^+^ T cells, inducing functional suppression. IL-11 overexpression promotes tumor progression and T cell dysfunction, whereas IL-11 knockdown reverses lactate-induced CD8^+^ T cell exhaustion, and cholesterol-modified siIL11 restores cytotoxic activity, improving immunotherapy efficacy (42).

Id2, a DNA-binding inhibitor, plays a critical role in regulating Tex. Id2 controls the generation of Tex^prog^ cells and their transition to Tex^term^ through transcriptional and epigenetic mechanisms (43). Genetic deletion of Id2 impairs CD8^+^ T cell-mediated immune responses, reduces stem-like CD8^+^ T cell maintenance (43), diminishes anti-PD-1 efficacy, and increases tumor susceptibility. Mechanistically, Id2 binds via its HLH domain to disrupt the Tcf3–Tal1 transcriptional complex, preventing Tcf3 interaction with histone lysine demethylase LSD1, thereby regulating chromatin accessibility at the Slamf6 promoter. This increases activating H3K4me2 marks at Tcf3-occupied E-box sites, promoting Tex^prog^ cell generation (43). LSD1 inhibition (GSK2879552) rescues the Id2-deficient phenotype in tumor models (44), expanding Slamf6^+^Tim-3^-^ Tex^prog^ cells and enhancing Tcf1 expression in Id2-deficient CD8^+^ T cells (45). Histone deacetylase inhibitors have been shown to modulate myeloid cell types, enhance tumor antigen presentation, and upregulate chemokines, thereby promoting antitumor T cell immunity. Clinical-stage histone deacetylase is profoundly impact gene expression and signaling networks in CD8^+^ and CD4^+^ T cells, enhancing effector functions including TNF-α and IFN-γ production and CD8^+^ cytotoxicity. In murine tumor models, histone deacetylase is enrich CD8^+^ T cell subsets expressing high levels of effector molecules (Prf1, Ifng, Gzmk, Gzmb) while also upregulating exhaustion-associated molecules (*Tox, Pdcd1, Lag3, Havcr2*), shaping a tumor microenvironment dominated by immunosuppressive myeloid cells. CD8^+^ T cell exhaustion, driven by chronic antigen stimulation, diminishes disease control (37). Both *in vitro* and *in vivo* models reveal global increases in repressive histone mark H3K27me3 in exhausted CD8^+^ T cells, accompanied by upregulation of the PRC2 complex subunit EZH2. H3K27me3 enrichment correlates with transcriptional downregulation, particularly at naive/memory-associated loci. PRC2 inhibition enhances naive/memory gene expression while reducing key exhaustion gene expression (46). Enhancer elements and transcription factors potentially regulating PRC2 subunit expression have been identified. Focusing on chromatin remodelers, *in vivo* CRISPR screens of protein domains reveal distinct roles for two SWI/SNF complex forms in Tex differentiation: canonical BAF loss impairs initial responses in acute and chronic infections, whereas PBAF (Both are subtypes of the mammalian SWI/SNF (BAF) chromatin-remodeling complex. PBAF (Polybromo-associated BAF) contains unique subunits such as PBRM1, ARID2, and BRD7, functioning mainly in enhancer regulation and transcriptional repression. Canonical BAF (cBAF) includes ARID1A/B and DPF2, often associated with transcriptional activation and maintenance of open chromatin) disruption enhances Tex proliferation and survival (47). Mechanistically, PBAF regulates the epigenetic and transcriptional transition of TCF-1^+^ precursor Tex to more differentiated TCF-1^-^ Tex subsets, maintaining precursor identity, while BAF supports effector-like Tex generation. Targeting PBAF, alone or with anti-PD-L1 therapy, improves tumor control, highlighting its potential as a novel immunotherapeutic target (47). Collectively, these histone and chromatin modifications lock CD8^+^ T cells into a stable exhausted state, underscoring epigenetic regulation as a key target for restoring their antitumor function (**Figure 2**).

## 5 Transcription factors and CD8^+^ T cell exhaustion

The formation and maintenance of CD8^+^ Tex are highly dependent on specific transcription factors, which shape characteristic transcriptional programs by promoting the expression of exhaustion-associated genes while suppressing effector genes (48). TOX is a central regulator in exhausted CD8^+^ T cells, whose expression is induced by persistent antigen stimulation via the calcineurin–NFAT2 pathway and maintained through a positive feedback loop within Tex cells (49). TOX not only drives high-level expression of exhaustion-associated genes but also remodels chromatin, thereby stabilizing the Tex state. ETS-family transcription factor Fli1 restricts Teff differentiation by binding cis-regulatory elements of effector genes. Fli1 deletion enhances Teff responses without affecting memory or precursor Tex formation, suggesting a key role in maintaining Tex progenitor states and preventing excessive effector differentiation (49). CD137 signaling promotes the proliferation and terminal differentiation of Tex progenitor cells via RelA/cRel-dependent NF-κB pathways and TOX-mediated chromatin remodeling, whereas matrix Gla protein (MGP) activates PD-L1 expression through NF-κB, thereby promoting CD8^+^ T cell exhaustion (50). The NR4A family (NR4A1/2/3) and ETV7 are additional critical exhaustion transcription factors. NR4A, activated downstream of NFAT, regulates both CAR T cells and endogenous CD8^+^ T cells toward hyporesponsive states (51); NR4A deletion enhances effector functions and antitumor activity. ETV7 acts as a central transcriptional node, biasing transcriptional programs of memory and exhaustion genes toward terminal exhaustion. Loss of ETV7 improves antitumor efficacy of both CAR T cells and endogenous CD8^+^ T cells (51). Transcription factors closely cooperate with chromatin-regulating complexes: distinct SWI/SNF complexes (BAF and PBAF) regulate progenitor versus effector-like Tex differentiation, while TOX, NR4A, and ETV7 bind accessible chromatin to sustain exhaustion programs, highlighting extensive crosstalk between transcription factors, chromatin state, and histone modifications. The transcription factor Osr2 integrates biomechanical signals to promote the terminal exhaustion of tumor-specific CD8^+^ T cells. In the terminally exhausted subset of these cells, Osr2 expression is induced by the combined effects of TCR signaling and biomechanical stress mediated via the Piezo1/calcium/CREB axis (18). Functional experiments show that deletion of Osr2 alleviates the exhaustion of tumor-specific CD8^+^ T cells or CAR-T cells, whereas forced overexpression of Osr2 exacerbates CD8^+^ T cell exhaustion in solid tumor models. Mechanistically, Osr2 recruits histone deacetylase 3 to reprogram the epigenetic landscape, suppress cytotoxic gene expression, and thereby promote CD8^+^ T cell exhaustion (18).

A subset of TCF-1^+^ CD8^+^ T cells exhibits stem-like properties, including self-renewal, proliferation, and differentiation into exhausted T cells (52). In murine LCMV clone 13 infection and tumor models, PD-1 antibody therapy further enhances these capabilities. These stem-like T cells, also termed progenitor exhausted T cells, express high levels of CXCR5, SLAMF6, and TCF-1 (53). TCF-1 expression is critical for the formation and maintenance of this progenitor subset. Early during chronic viral infection, TCF-1 antagonizes terminal effector formation and promotes progenitor Tex development. Upon anti-PD-1 treatment, progenitor Tex cells constitute the primary source differentiating into terminally exhausted T cells (53). TCF-1 cooperates with additional transcription factors to orchestrate multi-layered differentiation from progenitor to terminal Tex, ultimately locking T cells in a persistent dysfunctional state and limiting antiviral and antitumor responses. However, the molecular mechanisms governing the maintenance of stem-like features and differentiation toward exhaustion in TCF-1^+^ cells remain largely undefined (21). In hCD19^+^ tumor-bearing mice, transferred hCD19-specific CAR T cells reveal that tumor-infiltrating CD8^+^ CAR^+^ and endogenous CD8^+^ T cells co-expressing PD-1 and TIM3 share similar transcriptional and chromatin accessibility profiles, correlating with NFAT-driven secondary activation of NR4A1/2/3 (NUR77/NURR1/NOR1) (21). In patients with cancer or chronic infection, CD8^+^ T cells similarly show high NR4A expression and enrichment of NR4A binding motifs in accessible chromatin. Triple NR4A-deficient CAR T cells promote tumor regression, prolong survival, and exhibit effector-like CD8^+^ T cell phenotypes (48). Compared with wild-type, tumor-infiltrating lymphocytes in triple NR4A-deficient CAR T cells display open chromatin enriched for NF-κB and AP-1 motifs, which are involved in T cell activation, highlighting the critical role of NR4A in hyporesponsive programs and suggesting NR4A inhibition as a potential immunotherapeutic strategy (48). Tex cells exhibit restricted effector function, high inhibitory receptor expression, and broad transcriptional reprogramming compared to Teff and Tmem. They are major clinical targets for ICIs and other immunotherapies (54). From an epigenetic perspective, Tex cells represent a unique immune subset with chromatin landscapes distinct from Teff and Tmem cells. HMG-box transcription factor TOX is central to Tex formation in mice, while it has minimal impact on Teff and Tmem development (54). TOX deficiency prevents Tex formation. Induced by calcineurin–NFAT2 signaling, TOX establishes a positive feedback loop within Tex cells, maintaining expression independent of calcineurin. High TOX expression converts persistent stimulation signals into Tex-specific transcriptional and epigenetic programs, committing cells to exhaustion. CD137 (4-1BB), a potential immunotherapy target, regulates PD-1, LAG-3, and TIM-3 expression in tumor-infiltrating Tex cells. Acting independently of TCR signaling, CD137 stimulates progenitor Tex proliferation and terminal differentiation via RelA/cRel NF-κB subunits and TOX-dependent chromatin remodeling (54). Preventive CD137 agonist administration accumulates Tex cells and promotes tumor growth, but subsequent anti-PD-1 therapy significantly enhances efficacy (55). MGP is upregulated in primary colorectal cancer (CRC) and liver metastases, where it increases intracellular free Ca²^+^ and NF-κB phosphorylation, activating PD-L1 and promoting CD8^+^ T cell exhaustion (56). Luciferase and ChIP-qPCR assays confirm NF-κB transcriptional regulation of PD-L1. MGP inhibition in murine liver metastasis models reduces CRC metastasis and synergizes with anti-PD-1 therapy. Terminal exhaustion is a major barrier to antitumor immunity.

CD8^+^ T cells play a critical role in antitumor immune responses, yet the clinical efficacy of immunotherapy remains limited in many solid tumors. Using an epigenome-wide CRISPR-Cas9 screen directly in CD8^+^ T cells under a cancer immunotherapy context, we identified Prdm12 as a transcriptional repressor. Deletion of Prdm12 markedly enhanced *in vivo* tumor clearance by mouse CD8^+^ T cells and promoted activation, effector differentiation marker expression, and cytokine secretion in both murine and human CD8^+^ T cells *in vitro*. Mechanistically, Prdm12 deficiency augmented effector transcriptional programs while suppressing exhaustion signals mediated by the CGRP-RAMP1 neuroimmune axis. Furthermore, Prdm12 ablation remodeled the chromatin accessibility landscape, with H3K9me3 deposition at loci regulating T cell differentiation (Trib1, Sgk1) and exhaustion (Rgs1, Nr4a2) (57). T cell receptor (TCR) activation is regulated by multiple mechanisms, including niche-specific nutrient availability. We investigated how methionine (Met) availability interacts with TCR signaling during early T cell activation to influence cell fate. Limiting Met during the first 30 minutes of TCR engagement increased Ca²^+^ influx, NFAT1 (encoded by Nfatc2) activation, and promoter occupancy, leading to T cell exhaustion. Proteomic analyses revealed dynamic changes in arginine methylation during early TCR signaling, and identified arginine methylation of the Ca²^+^-activated potassium channel KCa3.1 as a key regulator of Ca²^+^-mediated NFAT1 signaling for optimal activation (58). Ablation of KCa3.1 arginine methylation enhanced NFAT1 nuclear localization and impaired T cell function in mouse tumor and infection models. Notably, acute early Met supplementation reduced nuclear NFAT1 in tumor-infiltrating T cells and enhanced antitumor activity (58). The transcription factor TOX initiates Tex cell epigenetic programming, yet whether TOX continues to maintain Tex identity after cell fate establishment remains unclear. Here, inducible deletion of TOX in committed Tex cells caused apoptotic loss, reduced inhibitory receptor expression, and decreased terminal differentiation. Gene expression and epigenetic profiling revealed that TOX is critical for maintaining chromatin accessibility and transcriptional programs in established Tex cells (59). Moreover, TOX ablation conferred greater fate plasticity, allowing Tex cells to differentiate into more functional effector-like T cells. Thus, continuous TOX expression acts as a durable epigenetic barrier reinforcing Tex developmental fate, and manipulation of TOX even after Tex formation may provide therapeutic opportunities to reprogram Tex cells in chronic infection or cancer (59). Integration of scRNA-seq and scATAC-seq data reveals that ETV7 is indispensable for determining CD8^+^ T cell fate in tumors (51). ETV7 drives memory T cells toward terminal exhaustion, limiting antiviral and antitumor responses. Mechanistically, ETV7 serves as a central node, directing transcriptional programs of memory and exhaustion genes toward exhaustion. Clinically, ETV7 expression negatively correlates with disease progression and ICI responsiveness in multiple human cancers (51). ETV7 deletion enhances the antitumor efficacy of CD8^+^ T cells and engineered CAR T cells in solid tumors. Chromatin accessibility analyses of HCV- and HIV-specific CD8^+^ T cells identified core epigenetic programs of exhaustion, which undergo limited remodeling pre- and post-infection (51). Exhaustion hallmarks, such as TOX and HIF1A-associated super-enhancers, maintain “epigenetic scars.” Hence, T cell exhaustion represents a conserved epigenetic state, once established, fixed and persistent, independent of chronic antigen or inflammation. Strategies to reverse exhaustion may require approaches that increase the epigenetic plasticity of exhausted T cells. Overall, transcription factors orchestrate the epigenetic and transcriptional programs that drive CD8^+^ T cell exhaustion, highlighting their potential as targets to reinvigorate antitumor immunity (**Figure 2**).

## 6 RNA epigenetic modifications and CD8^+^ T cell exhaustion

Beyond DNA- and histone-level epigenetic regulation, RNA epigenetic modifications play a pivotal role in the formation and maintenance of CD8^+^ Tex (60). Major RNA modifications include N6-methyladenosine (m6A), 5-methylcytosine (m5C), and pseudouridine (Ψ), which fine-tune gene expression by regulating mRNA stability, splicing, transport, and translation efficiency (60, 61). In Tex cells, m6A modifications significantly influence the fate of mRNAs encoding exhaustion-associated transcription factors and signaling molecules. Additionally, RNA modifications can indirectly modulate T cell function by affecting the expression of metabolism-related or immunosuppressive genes. For example, within the tumor microenvironment, RNA modifications may enhance inhibitory receptor expression or suppress effector cytokine production, thereby accelerating Tex formation and stabilizing the exhausted state (61). These RNA-level modifications cooperate with DNA methylation, histone modifications, and chromatin remodeling to establish the multilayered epigenetic landscape characteristic of Tex cells, conferring stability and resistance to reversal (62). From a therapeutic perspective, targeting RNA modifications presents novel opportunities to improve CD8^+^ T cell function. Modulating m6A methyltransferases or demethylases can alter the fate of key exhaustion-related mRNAs, potentially enhancing the efficacy of ICIs or CAR T cell therapy. This emerging field highlights the critical role of RNA epigenetic regulation in Tex cells, offering new strategies to reverse exhaustion and enhance antitumor immunity (10, 14, 39, 54, 63–66). In hepatocellular carcinoma, WTAP, an m6A methyltransferase, is upregulated in tumor-infiltrating CD8^+^ T cells (67). WTAP increases m6A modification on PD-1 mRNA, promoting YTHDF1-mediated PD-1 translation, thereby suppressing CD8^+^ T cell proliferation and immune activity, leading to exhaustion and enhanced tumor malignancy. WTAP knockdown alleviates CD8^+^ T cell exhaustion and improves anti-PD-1 therapeutic efficacy, demonstrating that WTAP drives hepatocellular carcinoma progression and attenuates antitumor immunity via epitranscriptomic regulation of PD-1 translation (67). In systemic lupus erythematosus, a CD7^high^ CD74^high^ CD8^+^ T cell subset exhibits exhaustion characteristics and is markedly expanded in patients. The m5C methyltransferase NSUN4 maintains CD74 expression through m5C modification, promoting CD8^+^ T cell exhaustion via CD44/mTOR-mediated mitophagy (68). NSUN4 knockdown reduces CD74 expression, suppresses exhaustion, and alleviates autoimmune responses and renal damage, indicating that NSUN4-mediated m5C modifications of mitophagy are critical for CD8^+^ T cell dysfunction in systemic lupus erythematosus pathogenesis (68). In non-small cell lung cancer NSUN2 and its reader protein ALYREF are upregulated in tumor cells and enhance PD-L1 mRNA stability through m5C modification, leading to PD-L1 overexpression (69). Elevated PD-L1 suppresses CD8^+^ T cell activation and infiltration, promoting immune evasion. NSUN2 knockdown reduces m5C modification and stability of PD-L1 mRNA, enhancing CD8^+^ T cell-mediated antitumor immunity, demonstrating that the NSUN2/ALYREF/PD-L1 axis directly mediates immunosuppression via m5C modification and drives non-small cell lung cancer progression (69). In CRC, YWHAH is stabilized and upregulated via N4-acetylcytidine modification, promoting tumor progression. High YWHAH expression is associated with immune evasion and poor prognosis, driving CD8^+^ T cell exhaustion, characterized by reduced proliferation and increased exhaustion markers (70). NAT10-mediated N4-acetylcytidine modification is critical for YWHAH stabilization, highlighting the central role of epitranscriptomic regulation in modulating the tumor microenvironment and immune escape, and identifying YWHAH as a potential immunotherapeutic target in CRC (70). “In summary, RNA epigenetic modifications critically shape the fate and function of CD8^+^ T cells, contributing to exhaustion and representing promising targets for restoring antitumor immunity (**Figure 2**).

## 7 Non-coding RNAs and CD8^+^ T cell exhaustion

Non-coding RNAs (ncRNAs) play crucial regulatory roles in the formation and maintenance of CD8^+^ Tex. NcRNAs primarily include microRNAs (miRNAs), long non-coding RNAs (lncRNAs), and circular RNAs, which modulate post-transcriptional gene expression, influence signaling pathway activity, or affect epigenetic states to contribute to the establishment of exhaustion (71). In Tex cells, multiple miRNAs directly target mRNAs encoding exhaustion-associated transcription factors or inhibitory receptors. For instance, specific miRNAs regulate the expression of TOX, the NR4A family, PD-1, or TIM-3, thereby influencing the initiation and maintenance of the exhaustion program (72–75). LncRNAs interact with transcription factors, chromatin remodeling complexes, or epigenetic enzymes to regulate chromatin accessibility and transcriptional activity of key genes, contributing to the differentiation and functional maintenance of exhausted T cell subsets (76, 77). circular RNAs act as “miRNA sponges,” competitively binding miRNAs and indirectly modulating the expression of exhaustion-related genes. NcRNAs closely interact with DNA methylation, histone modifications, and RNA epigenetic modifications, collectively establishing a multilayered epigenetic landscape in Tex cells, which stabilizes the exhausted phenotype and renders it difficult to reverse (78). Therapeutically, targeting ncRNAs shows potential to restore CD8^+^ T cell effector function and enhance the efficacy of ICIs or CAR T cell therapy.

Persistent viral infections and tumors induce Tex formation, maintaining a transient equilibrium between host and pathogen or tumor. Over time, Tex cells lose function, leading to pathogen or tumor escape. MiR-155 has been identified as a key regulator in maintaining Tex responses during chronic LCMV infection (79): miR-155 deficiency impairs CD8^+^ T cell responses, whereas overexpression promotes Tex expansion and long-term survival, although it does not fully reverse exhaustion but favors terminal Tex subset formation (80). Transcriptomic analyses indicate that miR-155 coordinates cell signaling and transcription factor pathways, with AP-1 family member Fosl2 acting as a critical mediator (81); Fosl2 overexpression can counteract miR-155 (79), suggesting that miR-155 regulates Tex cells via modulation of the AP-1 transcriptional program. MiR-29a is another critical regulator of Tex. Forced expression of miR-29a improves CD8^+^ T cell responses during chronic viral infection and counteracts exhaustion (82). Mechanistically, miR-29a suppresses exhaustion-driving transcriptional pathways (82), including inflammatory and TCR signaling, and regulates ribosome biogenesis, promoting memory-like differentiation and maintaining favorable functional states in chronic infection. Similarly, let-7 family miRNAs influence CD8^+^ T cell fate: maintaining let-7 expression during early activation promotes memory T cell formation and efficient tumor clearance, whereas let-7 deficiency drives terminal effector differentiation, predisposing cells to exhaustion and death in immunosuppressive environments (83). Let-7 exerts its effects by inhibiting PI3K/AKT/mTOR signaling and reactive oxygen species generation, limiting metabolic changes during activation, and preventing terminal differentiation and exhaustion. In human malignancies, ncRNAs are also implicated in immune escape (83). For instance, FOXP4-AS1 is highly expressed in esophageal cancer (EC), correlating with reduced tumor-infiltrating cytotoxic T lymphocytes and increased PD-L1 expression, leading to cytotoxic T lymphocyte apoptosis and functional suppression (84). Mechanistically, FOXP4-AS1 binds the deubiquitinase USP10, stabilizing PD-L1 and promoting CD8^+^ T cell exhaustion, thereby facilitating tumor growth and immune evasion. In hepatocellular carcinoma, tumor-derived extracellular vesicles (EVs) enriched with TGFβ-induced lncRNA HDAC2-AS2 suppress cytotoxic CD8^+^ T cell activity. HDAC2-AS2 targets cytoplasmic CDK9, disrupting its nuclear-cytoplasmic translocation and promoting PD-1^+^ CD8^+^ T cell exhaustion, while inhibiting IFN-γ^+^ CD8^+^ T cell cytotoxicity (84). Low CDK9 and high HDAC2-AS2 expression correlate with poor prognosis, which can be improved by anti-PD-1 therapy (77). Lnc-Tim3 is upregulated in tumor-infiltrating CD8^+^ T cells from hepatocellular carcinoma patients, negatively correlating with IFN-γ and IL-2 production. Lnc-Tim3 binds Tim-3, blocking its interaction with Bat3, thereby inhibiting downstream Lck/NFAT1/AP-1 signaling, retaining Bat3 in the nucleus, and enhancing p300-dependent transcriptional activation of anti-apoptotic genes (e.g., MDM2, Bcl-2), promoting CD8^+^ T cell exhaustion and survival (85).

CircRNAs also modulate Tex formation. In bladder cancer, exosomal hsa_circ_0085361 (circTRPS1) sponges miR-141-3p, regulating glutamine metabolism (GLS1 pathway) and tumor cell invasion and proliferation. Knockdown of circTRPS1 in exosomes prevents CD8^+^ T cell exhaustion and suppresses malignancy. Similarly, gastric cancer small extracellular vesicles carry circ_0001947, which sponges miR-661 and miR-671-5p, upregulating CD39 and accelerating CD8^+^ T cell exhaustion, while blockade enhances anti-PD-1 therapy (86). In pancreatic cancer, hsa_circ_0046523 is upregulated, correlating with advanced stage and poor prognosis. Overexpression promotes pancreatic cancer cell proliferation, migration, and invasion, while co-culture experiments show reduced CD4^+^/CD8^+^ T cell proportions, enhanced apoptosis, and increased IL-10/TGF-β with decreased IFN-γ/IL-2. Mechanistically, hsa_circ_0046523 sponges miR-148a-3p, increasing PD-L1 expression and promoting immune suppression (87). Collectively, non-coding RNAs regulate the transcriptional and post-transcriptional programs that drive CD8^+^ T cell exhaustion, offering potential targets to enhance antitumor immune responses.

## 8 Epigenetic plasticity and therapeutic intervention

Although CD8^+^ T cell exhaustion is generally considered a “locked” epigenetic state, recent studies indicate that Tex cells retain a degree of epigenetic plasticity, particularly in early or progenitor-like exhausted T cell populations (88). Single-cell multi-omics approaches—including scATAC-seq, single-cell DNA methylation, and transcriptome sequencing—have revealed heterogeneity within exhausted T cells. Progenitor-like Tex cells (TCF1^+^/PD-1^+^) maintain partially open chromatin and reversible enhancer/promoter regions, retaining limited proliferative and differentiation potential (88). In contrast, terminally exhausted T cells (PD-1^hi^/TOX^hi^) exhibit highly closed chromatin, restricted effector gene expression, and nearly irreversible epigenetic states (89). This hierarchical structure suggests that exhaustion is a dynamic process, with specific subsets amenable to functional restoration, providing a rationale for precision therapeutic strategies. Building on this plasticity, epigenetic drugs have emerged as promising tools to reverse exhaustion and enhance immune function (89). Current approaches include inhibition of DNA methyltransferases to reduce methylation at effector gene promoters and enhancers, restoring transcription of key molecules such as IFN-γ and GZMB while partially suppressing stable expression of inhibitory genes like *PDCD1 (* 90). Histone deacetylase inhibitors modulate histone acetylation, open closed chromatin regions, enhance effector gene transcription, and improve accessibility of metabolic genes, thereby boosting Tex cytotoxicity and proliferative capacity. Targeting enhancer function can reprogram exhaustion-related transcriptional networks, reduce the activity of exhaustion-driving transcription factors, and restore partial effector function (90). To highlight the translational potential, we provide a concise summary of epigenetic drugs currently in clinical trials, including their molecular targets, clinical status, and combination strategies (**Table 3**).

**Table 3:** Clinical trials of epigenetic modulators targeting exhausted CD8^+^ T cells and combination strategies.

Drug	Epigenetic target	Clinical trial status	Combination strategy	Notes
Azacitidine	DNA methyltransferase inhibitors	Phase II/III	With anti-PD-1 (nivolumab)	Restores effector gene expression in T cells
Decitabine	DNA methyltransferase inhibitors	Phase I/II	With anti-CTLA-4	Reprograms progenitor-like Tex cells
Vorinostat (SAHA)	histone deacetylase inhibitor	Phase I/II	With ICIs or chemotherapy	Enhances chromatin accessibility and cytokine production
Romidepsin	histone deacetylase inhibitor	Phase I/II	With anti-PD-1	Improves T cell proliferation and cytotoxicity
Tazemetostat	EZH2 inhibitor	Phase II	With ICIs	Modulates H3K27 methylation, reactivates effector genes

Clinically, combining epigenetic therapy with ICIs demonstrates considerable potential. ICIs alone (e.g., anti-PD-1 or anti-CTLA-4 antibodies) can relieve some inhibitory signaling but show limited efficacy in restoring terminally exhausted T cell function (91). Epigenetic interventions can reopen critical effector gene chromatin, recalibrate transcription factor networks, and enhance metabolic adaptability, thereby improving Tex responsiveness to ICIs (3). Preclinical models and early clinical data indicate that this combinatorial strategy enhances anti-tumor immunity, increases cytokine secretion and cytotoxicity, reduces tumor burden, and prolongs survival (92, 93). In summary, the epigenetic plasticity of exhausted T cells provides a therapeutic window for intervention. By precisely identifying Tex subsets, reprogramming T cell states with epigenetic modulators, and combining with checkpoint blockade, it is possible to achieve partial or stage-specific restoration of T cell function (3), significantly enhancing antiviral and antitumor immunity. This approach offers a conceptual and practical framework for future personalized immunotherapy and engineered T cell design.

Despite the promising potential of epigenetic therapies, several limitations and risks must be considered. Broad inhibition of DNA methyltransferases or histone deacetylase can lead to off-target effects, including global changes in gene expression that may cause toxicity, impair hematopoiesis, or disrupt the identity and function of non-target immune cells. Moreover, excessive or untargeted epigenetic reprogramming could inadvertently activate oncogenic pathways or promote T cell overstimulation, leading to exhaustion or apoptosis. Achieving selective modulation of exhausted T cell subsets remains challenging, and current pharmacological agents may not fully discriminate between progenitor-like and terminal Tex cells. Therefore, while epigenetic interventions offer a powerful tool to restore T cell function, careful optimization of dosing, timing, and combinatorial strategies is essential to maximize therapeutic benefit while minimizing adverse effects.

Recent clinical trials have begun to translate these epigenetic insights into therapeutic practice. DNMT inhibitors such as azacitidine and decitabine are being evaluated in combination with PD-1 blockade in solid tumors and hematologic malignancies (e.g., NCT04250246, NCT02961101), showing preliminary evidence of enhanced CD8^+^ T cell infiltration and reinvigoration. Similarly, HDAC inhibitors (e.g., entinostat, vorinostat) have demonstrated synergistic effects with ICIs in melanoma and non-small cell lung cancer by remodeling chromatin accessibility and increasing IFN-γ production. Other innovative approaches target BET proteins or EZH2 to suppress exhaustion-driving transcriptional programs (e.g., NCT04705818), representing a new generation of selective epigenetic modulators. Nevertheless, selective targeting of exhausted T cell subsets remains a major challenge. Most current epigenetic drugs act systemically, affecting multiple immune and non-immune lineages. This lack of specificity can perturb normal hematopoiesis, alter macrophage or dendritic cell function, or even trigger oncogenic reactivation. To mitigate such off-target risks, emerging strategies include: (1) nanoparticle-based delivery systems that restrict drug distribution to T cells within the tumor microenvironment; (2) prodrug formulations activated by tumor-specific enzymes or metabolic conditions; (3) CRISPR/dCas9-based epigenetic editing, enabling locus-specific modulation of exhaustion-related genes (e.g., *PDCD1, TOX*); and (4) temporal control of therapy, aligning epigenetic modulation with specific T cell differentiation windows to preserve progenitor pools. Future progress will depend on integrating multi-omic profiling and single-cell tracking to precisely define when and where epigenetic interventions are most effective. Ultimately, the development of selective, context-dependent epigenetic therapeutics will be key to safely reprogramming exhausted T cells while maintaining systemic immune balance. For clarity and consistency, **Table 4** lists all abbreviations used throughout this manuscript.

**Table 4:** List of abbreviations used in this manuscript.

Abbreviations	Full name
CD8^+^ T	CD8^+^ T cell
TCR	T cell receptor
TME	Tumor microenvironment
Tex	Exhausted T cell
Teff	Effector T cell
Tmem	Memory T cell
IR	Inhibitory receptor
PD-1	Programmed cell death-1
TIM-3	T cell immunoglobulin and mucin-domain containing-3
LAG-3	Lymphocyte activation gene-3
TIGIT	T cell immunoreceptor with Ig and ITIM domains
VISTA	V-domain Ig suppressor of T cell activation
CTLA-4	Cytotoxic T lymphocyte-associated protein-4
ICIs	Immune checkpoint inhibitors
m6A	N6-methyladenosine
m5C	5-methylcytosine
Ψ	Pseudouridine
DAC	Decitabine
GBP4	Guanylate-binding protein 4
ACSS2	Acetyl-CoA synthetase 2
ACLY	ATP-citrate lyase
Tex^prog^	Progenitor exhausted T cell
Tex^term^	Terminal exhausted T cell
H3K27me3	Histone H3 lysine 27 trimethylation
H3K4me3	Histone H3 lysine 4 trimethylation
H3K27ac	Histone H3 lysine 27 acetylation
PRC2	Polycomb repressive complex 2
SWI/SNF	SWItch/Sucrose Non-Fermentable
BAF	BRG1-associated factor
PBAF	Polybromo-associated BAF
HLH	Helix-loop-helix
CAR	Chimeric antigen receptor
scRNA-seq	Single-cell RNA sequencing
scATAC-seq	Single-cell Assay for Transposase-Accessible Chromatin sequencing
IFN-γ	Interferon-gamma
IL-2	Interleukin-2
TNF-α	Tumor necrosis factor-alpha
TCF-1	T cell factor 1
PD-L1	Programmed death-ligand 1
NSUN2	NOP2/Sun RNA methyltransferase family member 2
ALYREF	Aly/REF export factor
NAT10	N-acetyltransferase 10
EVs	Extracellular vesicles
lncRNA	Long non-coding RNA
miRNA	MicroRNA
circRNA	Circular RNA

## 9 Conclusion and future perspectives

Recent studies have highlighted the central role of epigenetic regulation in the initiation, maintenance, and functional impairment of CD8^+^ T cell exhaustion. Through coordinated remodeling of chromatin accessibility, DNA methylation, histone modifications, and transcription factor–epigenetic complexes, exhausted T cells establish a stable and specific epigenetic landscape. This framework not only explains the long-term stability and resistance to reversal of the exhausted state but also provides novel insights into the underlying mechanisms of T cell dysfunction.

Epigenetic interventions offer promising strategies to restore exhausted T cell function and enhance immunotherapy efficacy. Pharmacological agents, including DNA methyltransferase inhibitors, histone deacetylase inhibitors, and BET inhibitors, in combination with immune checkpoint blockade, can partially reverse exhaustion phenotypes, restore effector functions, and improve antiviral and antitumor immune responses (63). These approaches provide new avenues to overcome therapy resistance and functional decline, offering a theoretical basis for optimizing clinical immunotherapy. During chronic viral infections and tumor progression, dysregulated epigenetic modifications and cellular stress responses impair T cell immunity, representing a major factor in immunotherapy failure. Understanding how maladaptive metabolic programs and stress responses integrate with epigenetic changes during T cell exhaustion will expand insights into immunometabolic regulation of T cell differentiation. Exploring these under-investigated connections has the potential to reveal new therapeutic strategies for reactivating exhausted T cells and improving clinical outcomes of current immune checkpoint blockade treatments (94). Moreover, the metabolic pathways controlling T cell exhaustion intersect with systemic metabolic alterations caused by chronic infection, nutrient excess, and microbiota-derived metabolites. In the coming years, expanding our knowledge of how metabolic regulation and interventions influence T cell immunity will be critical for guiding future immunotherapeutic strategies.

Future research should focus on precise epigenetic targeting, synthetic biology approaches, and personalized immunotherapy. Leveraging single-cell and multi-omics technologies to finely map the epigenetic landscapes of exhausted T cell subsets enables dynamic stratification and precise intervention. Combined with gene-editing and epigenetic-modification tools, these strategies may allow the design of engineered T cells with enhanced resistance to exhaustion, thereby substantially improving therapeutic efficacy and safety. In summary, epigenetics provides both a conceptual and practical framework for understanding and reversing CD8^+^ T cell exhaustion, with broad prospects for application in personalized immunotherapy.

Despite these advances, several critical questions remain unresolved. One fundamental issue is the reversibility of epigenetic scars that stabilize the exhausted state. While early or progenitor-like Tex cells exhibit partial plasticity, terminally exhausted populations retain persistent DNA methylation and repressive chromatin configurations that are largely refractory to current therapeutic interventions. Whether these marks can be selectively erased without disrupting T cell identity or triggering aberrant activation remains an open question. Another emerging challenge concerns the role of tissue-specific cues in shaping exhaustion trajectories. The epigenetic and transcriptional programs of Tex cells differ markedly across tissues such as the tumor microenvironment, liver, and non-lymphoid sites, influenced by local cytokines, oxygen tension, antigen persistence, and stromal interactions. Understanding how these spatially defined signals imprint context-dependent epigenetic states will be crucial for designing tailored immunotherapies that operate effectively across diverse anatomical niches. Finally, the interplay between epigenetic regulation and metabolic state represents a rapidly evolving frontier. Metabolites such as acetyl-CoA, α-ketoglutarate, and S-adenosylmethionine serve as cofactors for chromatin-modifying enzymes, directly linking cellular metabolism to the establishment of exhaustion-related epigenetic programs. Dissecting how metabolic reprogramming can modulate epigenetic landscapes—and vice versa—may uncover strategies to metabolically “unlock” exhausted T cells. Addressing these unanswered questions will not only deepen our mechanistic understanding of T cell exhaustion but also pave the way for the rational design of stage-specific, tissue-adapted, and metabolism-informed epigenetic therapies, bringing precision immunomodulation closer to clinical realization.
